# Intimate partner violence, associations with perceived need for help and health care utilization: a population-based sample of women in Sweden

**DOI:** 10.1177/1403494820930952

**Published:** 2020-08-27

**Authors:** Solveig Lövestad, Marjan Vaez, Jesper Löve, Gunnel Hensing, Gunilla Krantz

**Affiliations:** 1 School of Public Health and Community Medicine, Sahlgrenska Academy at University of Gothenburg, Göteborg, Sweden; 2 Department of Clinical Neuroscience, Division of Insurance Medicine, Karolinska Institutet, Stockholm, Sweden

**Keywords:** Intimate partner violence, women, population-based, perceived need, health care utilization

## Abstract

*Aim:* To assess the association between physical intimate partner violence (physical IPV) in the past 5 years, perceived need for help and primary health care utilization due to mental health problems in a general population-based sample of women in Sweden. *Methods:* We performed structured follow-up interviews with 616 women between 1995 and 2015. Associations between physical IPV in the past 5 years and (i) perceived need for help and (ii) primary health care utilization due to mental health problems, were estimated by logistic regression analyses with crude and adjusted odds ratios (ORs) with 95% confidence intervals (CIs). *Results:* Of the women who had experienced physical IPV in the past 5 years, 45.1% perceived a need for help but refrained from seeking care. After adjusting for sociodemographic factors, exposure to physical IPV in the past 5 years remained associated with perceived need for help (OR 3.54; CI 1.77–7.11). After adjusting for sociodemographic factors, the association between exposure to physical IPV and primary health care utilization did not remain statistically significant. ***Conclusions:* Women exposed to physical IPV were more likely to perceive the need for help compared with unexposed women. A large proportion of IPV-exposed women in the general population may refrain from seeking care although they perceive a need for help. Future studies need to investigate potential barriers to mental health care seeking among women exposed to IPV. Routine questioning about IPV should be implemented in primary health care with improved referral to available support services.**

## Background

Intimate partner violence (IPV) perpetrated by men against women is a substantial public health concern encompassing physical, sexual and psychological violence [1]. A previous report based on the 28 member states of the European Union (EU) estimated that 12–31% of women in these countries had experienced physical IPV during their lifetime, perpetrated by a current or former partner [2].

Women experiencing physical IPV are at risk of mental health problems such as anxiety [2, 3], depressive symptoms [2, 4], psychological distress [5] and suicidal ideation and attempts [3]. These problems may persist over long periods, irrespective of whether the woman leaves the violent relationship or not [4]. Therefore, exposed women are more likely to use different health care services, including primary care [6] and mental health services [7]. Research from the United States shows that women reporting physical IPV are about three times more likely to use mental health services compared with those who are unexposed to such violence [8]. Exposed women are also more likely to be long-term users of mental health services, even after the violence has ceased [6]. Bonomi and colleagues [6] found that for women exposed to physical IPV within the past 5 years or before that, mental health care utilization was still higher as compared to women who never had experienced such violence. However, much of the current literature has been based on clinic samples [9] or convenience samples [10] and has been conducted in the United States [6, 8, 9]. To the best of our knowledge, there has been no previous population-based study performed in Sweden that has investigated health care utilization among women exposed to IPV.

Women exposed to IPV face several barriers to health care utilization [11-13] such as financial strain [13], self-blaming, fear of repercussions from the partner and low trust in health care professionals [11]. According to Andersen’s behavioural model [14], attitudes, values, and knowledge about health and health services, are important factors that influence subsequent perceived need and health care utilization. Self-perceived need refers to people’s views and whether they perceive their own health problem as sufficiently important as to seek professional health care [14]. Perceived need for help is therefore a crucial step before seeking care [14]. Previous research from the United States, using data from shelters, found that twice as many women exposed to IPV reported the need for mental health care compared with those reporting the need for physical health care (40.4% and 19.2% respectively) [15]. To date, there has been a lack of population-based studies exploring the association between exposure to IPV, perceived need for help and primary health care utilization within a Swedish context. Population-based studies include women outside the health care settings and may therefore contribute to a more complete picture of the occurrence of IPV and its association with perceived need for help and health care utilization.

The purpose of the current study was to assess the association between self-reported exposure to physical IPV in the past 5 years, perceived need for help and primary health care utilization due to mental health problems, among women from a population-based sample in Sweden.

## Methods

This study consists of data from the Swedish, population-based study ‘Women and Alcohol in Gothenburg’ (WAG), which was initiated with the aim of improving knowledge about women’s alcohol consumption and mental disorders. WAG applies a two-stage, stratified random sampling design with the aim of increasing the recruitment of individuals with alcohol-related problems, while keeping the numbers of participants at a reasonable level [16].

### Stage 1: Screening

**Figure 1.**
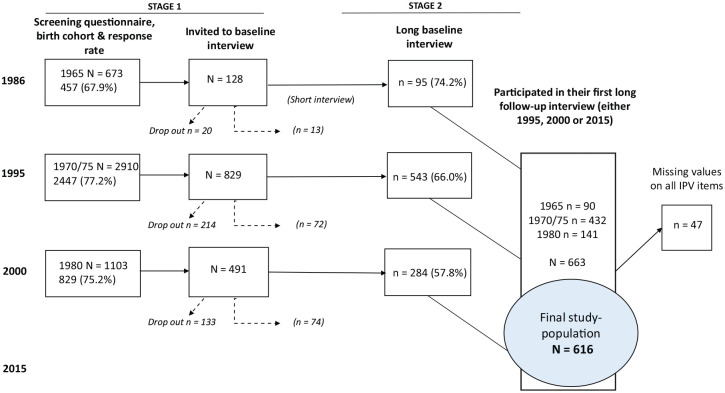
Flowchart of the data collection and final study population: *N* = 616.

In 1986, a questionnaire for the screening of alcohol-related problems was mailed out to all women born in 1965 (*N* = 673) who were registered in the central and western districts of Gothenburg. In 1995 the same procedure was applied regarding women born in 1970 and 1975 (*N* = 2910), and in the third wave in 2000, regarding women born in 1980 (*N* = 1103). The response rates at each screening wave are presented in Figure 1. The screening questionnaire contained 13 items where each positive answer scored 1 point, making the maximum total score 13 points. Based on the scores, the answers were grouped into three categories. All respondents with scores of ⩾5 (probable alcohol-related problems), a random quarter of those with 1–3 points and a random one-fifteenth of those who scored 0 points, were invited by letter to a face-to-face interview [17]. Written reminders were sent to non-responders and, if necessary, telephone calls were carried out.

### Stage 2: The interviews

The face-to face interviews were performed by a team of professionals in health care and social work, either at the respondent’s home or at the University of Gothenburg. Respondents who could not participate in a long face-to-face interview were offered a shorter version of the interview focusing on alcohol consumption and alcohol-related problems. Short follow-up interviews were not included in this particular study as they did not include any questions on perceived need for help, mental health care utilization or IPV. The interviews lasted for about 1.5–3 h. Out of those born in 1965, 74.2% of the invited women participated in a long baseline interview (Figure 1). In 1995, 66% of women born in 1970/1975 participated in a long baseline interview. In 2000, of those born in 1980, 57.8% participated in a long baseline interview.

### Study population

We used cross-sectional individual data from three waves of WAG. The pooled study sample consisted of 663 women who participated in their first long follow-up interview in 1994–1998, 2000–2002 or 2013–2015 (henceforth referred to as 1995, 2000 and 2015). Participants with missing values on all physical IPV items in their first follow-up interview (*n* = 47) were excluded from the dataset, thus leaving a final sample of 616 women (Figure 1).

### Outcome variables

‘Perceived need for help’ was assessed with the question, ‘During the past 5 years, was there any time when you felt so mentally distressed that you needed to seek help, or have you ever felt so mentally distressed that you could have benefitted from seeking help?’

*‘*Primary health care utilization due to mental health problems’ was assessed using the question, ‘Have you had any primary health care contact due to mental health problems in the past 5 years?’ Response options for each of the questions were binary (yes/no), with ‘no’ being the reference category in the bi- and multivariable logistic regression analyses.

### Exposure variables

IPV was considered to be the main exposure variable. In 1995 and 2000, physical IPV was measured by Straus’s original Conflict Tactics Scale (CTS1) [18]. The participants were asked whether their partner had perpetrated physical violence during the past 5 years (Table I). The questions were combined creating a dummy variable with an affirmative response to any of the four questions coded as 1 (exposure to physical partner violence ⩾1 time in the past 5 years). A negative response to all four questions was coded with the value zero (no exposure to physical partner violence). If missing values in all four variables, it was coded as ‘missing’ in the dummy variable.

**Table I.** Exposure to physical intimate partner violence (IPV) during past 5 years or earlier in life. Women participating in their first follow-up interview in 1995, 2000 or 2015 (*N* = 616).

Exposure to physical IPV past 5 years	1995		2000		Exposure to physical IPV earlier in life	2015^a^	
	*N* = 63		*N* = 349			*N* = 204	
	*n*	%^b^	*n*	%^b^		*n*	%^b^
Threw something at her					Slapped you or thrown something at you that could hurt		
Never	59	94.4	334	96.4	No	178	86.7
1–2 times	2	2.8	9	2.1	Yes	26	13.3
⩾3 times	2	2.8	6	1.5	NA	NA	NA
Pushed, grabbed, shoved her					Pushed you or shoved you or pulled your hair		
Never	55	88.1	297	88.3	No	173	86.7
1–2 times	6	9.1	37	8.6	Yes	31	13.3
⩾3 times	2	2.8	15	3.1	NA	NA	NA
Kicked, bit or hit her with a fist					Hit you with a fist or with something else that could hurt you		
Never	57	88.8	335	96.7	No	196	96.7
1–2 times	5	9.8	7	1.3	Yes	8	3.3
⩾3 times	1	1.4	7	2.0	NA	NA	NA
Beat her up					Kicked or dragged you or beaten you up		
Never	61	97.2	341	97.9	No	196	96.9
1–2 times	1	1.4	2	0.3	Yes	8	3.1
⩾3 times	1	1.4	6	1.8	NA	NA	NA

a In 2015 the IPV questions as well as the time frame were changed.

b Weighted percentages.

In 2015, the IPV questions were changed to the World Health Organization’s Violence Against Women Instrument in order to include questions on psychological IPV and controlling behaviour [1]. However, as questions on psychological IPV and controlling behaviour were not included in the first two waves (1995 and 2000), this study focuses on physical violence only. The time frame was changed from ‘past 5 years’ to ‘earlier in life’ and the response options to each of the four questions changed to ‘yes’ and ‘no’ (Table I). The four questions were combined and further dichotomized with an affirmative response to any of the four questions coded as ‘yes’ (exposed). A negative response to each of the questions was coded as ‘no’ (unexposed), being the reference category in the bi- and multivariable analyses.

### Covariates

Age at first follow-up interview was categorized into three groups: 25–29, 30–35 and 36–48 years. Educational level was classified into three categories: compulsory school (⩽9 years), secondary education (10–12 years) and post-secondary education (>12 years). Relationship status was categorized into three groups: (1) married, cohabiting, registered partnership (‘married/cohabiting’) (2) divorced, separated and (3) widowed, single, never married, non-cohabiting partner (‘widow/single’). Current occupation was classified under (1) those who were working part-time or more (‘employed’), (2) homeworkers, unemployed, women who responded ‘not working because of other reasons’ as well as those on disability pension or sickness absence exceeding 3 months (‘unemployed’), and (3) those studying part-time or more (‘students’). Participants on parental leave were categorized based on their occupation prior to parental leave. Annual income before tax was categorized into four groups: (1) <100,000 Swedish Krona (SEK) (2) SEK100,000–199,999 (3) SEK200,000–300,000 and (4) >SEK300,000. In order to improve the statistical power in the bi- and multivariable logistic regression analyses, the variable was further dichotomized into ⩾SEK100,000 (reference category) and <SEK100,000 respectively.

## Statistical methods

As the material was oversampled with individuals who had indicated possible alcohol-related problems on the screening questionnaire, the prevalence rates, crude and adjusted odds ratios (ORs) and 95% confidence intervals (CIs) were calculated on weighted values. This approach has been used and described in previous studies based on WAG [16, 17]. Briefly, this means that those with no and low probability of alcohol-related problems, were up-weighted to adjust for the oversampled group with probable alcohol-related problems. Hence, any prevalence and associations are distributed according to the source population, thereby minimizing bias related to the oversampled group. The analyses were carried out in SPSS version 24.0 (IBM Corp., Armonk, NY) using the Complex Samples Plan, which takes into account weights for oversampling of specific groups [19].

Chi-square was used to test for differences (*p* ⩽ .05) between respondents versus non-respondents to any of the physical IPV items. Descriptive statistics were presented with unweighted total numbers (*N*), unweighted frequencies (*n*) and weighted prevalence (%). Chi-square was also used to test for differences (*p* ⩽ .05) in physical IPV by sociodemographic factors, perceived need for help and primary health care utilization. Logistic regression analyses were performed producing crude and adjusted ORs with 95% CIs to analyse associations between exposure to physical IPV and (a) perceived need for help and (b) primary health care utilization due to mental health problems. Multivariable models were adjusted for age at first follow-up interview (continuous variable) and year of wave at first follow-up interview (i.e. 1995, 2000 and 2015). The associations between physical IPV, perceived need for help and primary health care utilization, were adjusted for current occupation, annual income, and educational level as these variables are considered to be potential confounders [3, 7, 11, 20].

## Ethical approval and consent to participate

This study was approved by the Regional Ethics Review Board in Gothenburg in 18 October 2016 (Dnr: T930-16). Participants provided verbal informed consent and were informed of available helplines and support services.

## Results

There were no significant differences between those who responded versus did not respond to any of the physical IPV items regarding age (*p =* .831), educational level (*p =* .831), occupational status (*p =* .910) and yearly income before tax (*p =* .406).

Overall, 14.1% had experienced physical IPV at least once during the past 5 years (Table II). Those exposed were more frequently between 25 and 29 years old, divorced or separated, had a lower educational attainment, were students or had a yearly income before tax of less than 100,000 SEK (*p* < .0001). A higher proportion of exposed women than those unexposed reported a need for help (67.9% vs. 37.1%) and primary health care utilization due to mental health problems (37.2% vs. 22.9%) (*p* < .0001).

**Table II.** Sociodemographic characteristics, perceived need for help and health care utilization by physical intimate partner violence (IPV) during past 5 years, Total sample of women *N* = 616.

	Exposure to physical IPV past 5 years				Chi^2^ tests
	Frequencies (*n*), prevalence (%)				
Sociodemographic characteristics, and mental health care variables	Never *n* = 515 (85.9%)^a^		Yes ⩾1 time *n* = 101 (14.1%)^a^		
Age at interview	*n*	% ^a^	*n*	% ^a^	*p* value
25–29 years	153	25.0	37	38.4	<.0001*
30–35 years	303	63.9	51	46.4	
36–48 years	59	11.1	13	15.2	
Relationship status					
Married/cohabiting	378	73.3	59	61.8	<.0001*
Divorced/separated	68	12.7	22	24.0	
Widow/single	69	14.0	19	13.9	
Missing	–	–	1	0.3	
Educational level					
Post-secondary education (>12 years)	338	65.1	51	52.5	<.0001*
Secondary education (10–12 years)	164	33.2	42	40.6	
Compulsory school (⩽9 years)	13	1.6	8	6.9	
Missing	–	–	1	0.3	
Current occupation					
Paid employment	400	80.4	70	73.6	<.0001*
Student	75	11.4	25	21.7	
Unemployed/not in labour force	40	8.2	6	4.7	
Annual income before tax					
>SEK300,000	125	18.0	20	13.9	<.0001*
SEK200,000–300,000	104	25.2	16	19.5	
SEK100,000–199,999	164	35.6	29	32.0	
<SEK100,000	95	16.7	26	23.2	
Missing	27	4.5	10	11.5	
Perceived need for help during past 5 years					
No (*N* = 331)	297	62.2	34	32.1	<.0001*
Yes (*N* = 282)	215	37.1	67	67.9	
Missing	3	0.6	0	0.0	
Primary health care utilization for mental health problems during past 5 years					
No (*N* = 413)	354	73.8	59	62.8	<.0001*
Yes (*N* = 184)	142	22.9	42	37.2	
Missing	19	3.3	0	0.0	

a Weighted percentages.

* *p* < .05.

Of the women exposed to physical IPV in the past 5 years, 54.9% (unweighted *n* = 42) perceived a need for help and had accessed the primary health care for their mental health problems (not in table). Among those experiencing IPV, 45.1% (unweighted *n* = 25), perceived a need for help but had not sought care for their mental health problems.

Table III demonstrates that women exposed to physical IPV during the past 5 years had a higher OR regarding perceived need for help than women without such experience (OR 3.54; 95% CI 1.87–6.68). This association remained significant when adjusting for age, year of interview wave and current occupation in Model 1 (OR 3.94; 95% CI 2.06–7.53). In Model 2, after adjusting for age, interview wave and annual income before tax, women exposed to physical IPV during past 5 years had a 3.5 times higher OR of need for help (3.54; 95% CI 1.77–7.11) compared with unexposed women.

**Table III.** Crude and adjusted associations between physical intimate partner violence (IPV), sociodemographic factors and perceived need for help in the past 5 years. Frequency (*n*), odds ratios (ORs) and 95% confidence intervals (CIs).

Experienced physical IPV	Perceived need for help		Weighted OR and 95% CI		
	No *N* = 331	Yes *N* = 282	Crude	^b^Model 1	^c^Model 2
	*n*	*n*	^a^OR (95%CI)	^a^OR (95%CI)	^a^OR (95%CI)
No	297	215	1	1	1
Yes	34	67	3.54 (1.87–6.68)	3.94 (2.06–7.53)	3.54 (1.77–7.11)
Education level					
Post-secondary education (>12 years)	199	188	1		
Secondary education (10–12 years)	121	84	0.75 (0.46–1.23)		
Compulsory school (⩽9 years)	11	10	1.25 (0.44–3.60)		
Current occupation					
Paid employment	264	203	1		
Student	45	55	1.50 (0.86–2.63)		
Unemployed/not in labour force	22	24	2.66 (1.09–6.47)		
Annual income before tax					
⩾SEK100,000	265	191	1		
<SEK100,000	56	65	2.23 (1.29–3.86)		
Missing	10	26			

a Weighted odds ratios (ORs) and 95% confidence intervals (CIs).

b Model 1: adjusted for age at the time of first follow-up interview, the three data collection waves (1995, 2000, 2015) and current occupation.

c Model 2: adjusted for age at first follow-up interview and the three data collection waves (1995, 2000, 2015) and annual income before tax.

Women exposed to physical IPV had a higher OR (1.91; 95% CI 1.02–3.57) for primary health care utilization due to mental health problems as compared to unexposed women (Table IV). After adjustments for age, interview wave and educational level in Model 1, exposed women had a twofold higher OR for primary health care utilization (OR 2.11; 95% CI 1.09–4.08). In Model 2, after adjusting for age, year of interview wave and annual income before tax, the association between exposure to physical IPV and primary health care utilization did not remain statistically significant

**Table IV.** Crude and adjusted association between physical intimate partner violence (IPV), sociodemographic factors and primary health care utilization for mental health problems past 5 years. Frequency (*n*), odds ratios (ORs) and 95% confidence intervals (CIs).

Experienced physical IPV	Primary health care utilization for mental health problems		Weighted odds ratio (OR) and 95% confidence interval (CI)		
	No *N* = 413	Yes *N* = 184	Crude	^b^Model 1	^c^Model 2
	*n*	*n*	^a^OR (95%CI)	^a^OR (95%CI)	^a^OR (95%CI)
No	354	142	1	1	1
Yes	59	42	1.91 (1.02–3.57)	2.11 (1.09–4.08)	1.74 (0.91–3.31)
Education level					
Post-secondary education (>12 years)	240	134	1		
Secondary education (10–12 years)	159	43	0.51 (0.29–0.90)		
Compulsory school	14	7	0.68 (0.23–1.92)		
Current occupation					
Paid employment	317	136	1		
Student	69	31	1.23 (0.69–2.21)		
Unemployed/not in labour force	27	17	1.64 (0.64–4.22)		
Annual income before tax					
⩾SEK100,000	320	122	1		
<SEK100,000	78	41	1.93 (1.08–3.46)		
Missing	15	21			

a Weighted odds ratios (ORs) and 95% confidence intervals (CIs).

b Model 1: adjusted for age at the time of first follow-up interview, the three data collection waves (1995, 2000, 2015) and educational level.

c Model 2: adjusted for age at first follow-up interview and the three data collection waves (1995, 2000, 2015) and annual income before tax.

## Discussion

To the best of our knowledge, this is the first population-based study in Sweden addressing women’s exposure to physical IPV and its associations with perceived need for help and primary health care utilization due to mental health problems. Overall, 14.1% of the women had experienced physical IPV at least once in the past 5 years. This is in line with lifetime estimates of exposure to physical IPV presented in previous prevalence studies from Sweden [21, 22]. Consistent with earlier findings, women who were younger [23], students, divorced or separated [24], with lower educational attainment [23] and lower annual income [6, 23], reported higher rates of exposure to physical IPV than unexposed women.

The association between exposure to physical IPV during the past 5 years and perceived need for help for exposed women was more than threefold that of unexposed women. Our results are in line with previous research based on clinic and convenience samples, indicating that many women exposed to IPV perceive the need for help in terms of care [9, 15]. Our study showed that among women exposed to physical IPV who also perceived a need for help, the majority sought primary health care. This is in accordance with previous research from the United States and Canada showing high prevalence rates of general health care consumption among women exposed to IPV [13, 25]. For instance, in a US study, 62.6% of the women experiencing IPV had visited a general mental health provider [25]. However, we found that 45.1% of the exposed women with perceived need for help had not accessed any primary health care services. This finding is similar to previous research where 49.4% of women who perceived a need for help did not seek any care [9]. We were not able to examine the underlying reasons for why exposed women who perceived a need for help did not seek care. The violent partner may prevent women from seeking care through the use of control tactics or by instilling fear [11]. Women may also feel ashamed and embarrassed for being exposed, thus avoiding contact with health services [11]. Other examples include language barriers [11] and low financial resources [11, 13]. Most of the literature in this matter comes from the United States [11] and barriers faced by women may differ across countries. Therefore, a possible area for future research would be to investigate potential barriers to health care seeking among women exposed to IPV in Sweden. Many women who seek care, never share their experience of exposure to IPV [2]. However, women may be more likely to disclose their exposure if they are asked about violence [26] and therefore, routine questioning about IPV with improved referral to available support services should be implemented.

After adjusting for annual income before tax, the association between physical IPV and primary health care utilization due to mental health problems was no longer statistically significant. In contrast, most of previous research has shown that women exposed to IPV are more likely to use health care services compared with unexposed women [6, 9] whether adjusted [27] or not adjusted for income [6, 9].

### Methodological considerations

A strength of this study was the use of a general population-based sample. Further, in accordance with ethical recommendations for IPV research, the interviewers were clinically experienced, had extensive training and continuous supervision during the data collection [28, 29]. Nevertheless, there are certain limitations. The IPV questions were about events that had happened in the past 5 years and ‘earlier in life’ with a potential recall bias. Further, since questions on psychological, sexual IPV and controlling behaviour were included in the most recent data collection only, it was not possible to analyse different forms of IPV and associations with perceived need for help and primary health care utilization. Our findings have to be understood with this in mind, since physical IPV is often accompanied by other forms of IPV [1]. Finally, our study had a cross-sectional design and therefore we cannot conclude any causal relationship between IPV and perceived need for help.

High levels of alcohol-related problems have been found to be associated with dropout from follow-up studies [30]. Further, IPV is a sensitive topic, which may lead to under-reporting of such experiences [29]. This suggests a lower participation rate of women with alcohol-related problems as well as under-reporting of exposure to IPV. Therefore, prevalence as well as associations between IPV and outcome variables may be somewhat underestimated in this study.

## Conclusions

Women exposed to physical IPV were more likely to perceive the need for help compared with unexposed women. Our findings suggests targeted training for primary health care providers in routine questioning about exposure to IPV as well as referral to available support services. Future studies need to investigate potential barriers and promoting factors to mental health care seeking among exposed women in the general population.
